# The high-density lipoprotein cholesterol (HDL-C)-concentration-dependent association between anti-inflammatory capacity and sepsis: A single-center cross-sectional study

**DOI:** 10.1371/journal.pone.0296863

**Published:** 2024-04-11

**Authors:** Kai-Lee Chen, Ruey-Hsing Chou, Chun-Chin Chang, Chin-Sung Kuo, Jih-Hua Wei, Po-Hsun Huang, Shing-Jong Lin

**Affiliations:** 1 Institute of Clinical Medicine, National Yang Ming Chiao Tung University, Taipei, Taiwan; 2 Cardiovascular Research Center, National Yang Ming Chiao Tung University, Taipei, Taiwan; 3 Division of Cardiology, Department of Medicine, Taipei Veterans General Hospital, Taipei, Taiwan; 4 Department of Critical Care Medicine, Taipei Veterans General Hospital, Taipei, Taiwan; 5 Division of Endocrinology and Metabolism, Department of Medicine, Taipei Veterans General Hospital, Taipei, Taiwan; 6 Division of Cardiology, Department of Internal Medicine, Min-Sheng General Hospital, Taoyuan, Taiwan; 7 School of Medicine, National Defense Medical Center, Taipei, Taiwan; 8 Healthcare and Services Center, Taipei Veterans General Hospital, Taipei, Taiwan; 9 Taipei Heart Institute, Taipei Medical University, Taipei, Taiwan; 10 Division of Cardiology, Heart Center, Cheng-Hsin General Hospital, Taipei, Taiwan; University Hospital Zurich: UniversitatsSpital Zurich, SWITZERLAND

## Abstract

**Introduction:**

Known to have pleiotropic functions, high-density lipoprotein (HDL) helps to regulate systemic inflammation during sepsis. As preserving HDL-C level is a promising therapeutic strategy for sepsis, the interaction between HDL and sepsis worth further investigation. This study aimed to determine the impact of sepsis on HDL’s anti-inflammatory capacity and explore its correlations with disease severity and laboratory parameters.

**Methods and materials:**

We enrolled 80 septic subjects admitted to the intensive care unit and 50 controls admitted for scheduled coronary angiography in this cross-sectional study. We used apolipoprotein-B depleted (apoB-depleted) plasma to measure the anti-inflammatory capacity of HDL-C. ApoB-depleted plasma’s anti-inflammatory capacity is defined as its ability to suppress tumor necrosis factor-α–induced vascular cell adhesion molecule-1 (VCAM-1) expression in human umbilical-vein endothelial cells. A subgroup analysis was conducted to investigate in septic subjects according to disease severity.

**Results:**

ApoB-depleted plasma’s anti-inflammatory capacity was reduced in septic subjects relative to controls (VCAM-1 mRNA fold change: 50.1% vs. 35.5%; *p* < 0.0001). The impairment was more pronounced in septic subjects with than in those without septic shock (55.8% vs. 45.3%, *p* = 0.0022). Both associations were rendered non-significant with the adjustment for the HDL-C level. In sepsis patients, VCAM-1 mRNA fold change correlated with the SOFA score (Spearman’s *r* = 0.231, *p* = 0.039), lactate level (*r* = 0.297, *p* = 0.0074), HDL-C level (*r* = –0.370, *p* = 0.0007), and inflammatory markers (C-reactive protein level: *r* = 0.441, *p* <0.0001; white blood cell: *r* = 0.353, *p* = 0.0013).

**Conclusion:**

ApoB-depleted plasma’s anti-inflammatory capacity is reduced in sepsis patients and this association depends of HDL-C concentration. In sepsis patients, this capacity correlates with disease severity and inflammatory markers. These findings explain the prognostic role of the HDL-C level in sepsis and indirectly support the rationale for targeting HDL-C as sepsis treatment.

## Introduction

Sepsis, defined as life-threatening organ dysfunction caused by a dysregulated host response to infection, is the greatest challenge in critical care, contributing to one-fifth of deaths in this setting worldwide [1, 2]. With the failure of hundreds of clinical trials, the number of medications with the ability to counteract sepsis remains limited [3, 4]. A more comprehensive understanding of the pathogenesis of sepsis is needed for the identification of new therapeutic targets.

High-density lipoprotein (HDL), the scavenger of cholesterol, is known to play a role in the reverse cholesterol transport pathway [5]. It has pleiotropic functions that help to maintain circulatory hemostasis such as the regulation of the inflammatory response and liposaccharide binding, and an antioxidant effect [6–8]. Clinically, a drastic decline in the high-density lipoprotein cholesterol (HDL-C) level occurs during sepsis and can serve as a prognostic factor [9, 10]. Recent studies have revealed that an inhibitory variant of the human cholesteryl ester transfer protein (CETP) gene is associated with an increase in the plasma HDL-C concentration and better sepsis outcome, whereas a gain-of-function variant of this gene is associated with the opposite effect [11–13]. Given the success of a CETP inhibitor in improving the survival of septic mice [12, 14], further investigation of the interaction between HDL and sepsis is warranted.

The anti-inflammatory capacity of HDL, defined as its ability to suppress tumor necrosis factor-α (TNF-α)–induced vascular cell adhesion molecule-1 (VCAM-1) expression at the endothelial level [15], is important for the attenuation of endothelial dysfunction and systemic inflammation primarily through the blunting of transcriptional factors such as nuclear factor kappa-B [16–18]. Data on this capacity in patients with sepsis are lacking, however, as most studies have focused on its association with cardiovascular disease [15, 19–21]. Thus, the observation of this HDL functionality in a septic population and the determination of its clinical relevance are of interest. In this cross-sectional study, we used apolipoprotein-B depleted (apoB-depleted) plasma to measure HDL’s anti-inflammatory capacity. The aim of the study was to determine whether apoB-depleted plasma’s anti-inflammatory capacity is associated with the presence and/or severity of sepsis, and to explore its correlations with various laboratory and clinical parameters.

## Methods

### Study population and data collection

We randomly enrolled 80 patients aged ≥ 18 years with sepsis who were admitted to the medical intensive care unit (ICU) of Taipei Veterans General Hospital, a tertiary medical center in Taiwan, between May 2018 and June 2019. Sepsis was diagnosed according to the Society of Critical Care Medicine’s sepsis-3 criteria [2] as a sequential organ failure assessment (SOFA) score increase ≥ 2 within 24 hours in the presence of infection. In addition, we randomly enrolled 50 subjects admitted for scheduled coronary angiography (CAG) between May 2019 and Apr 2021, and without acute coronary syndrome, as controls. By detailed chart review, the remaining information on the enrolled patients was collected: age, sex, body mass index (BMI; kg/m^2^), history of smoking, and comorbidities [type 2 diabetes mellitus (T2DM), hypertension, atrial fibrillation (AF), gout, end-stage renal disease, coronary artery disease (≥50% stenosis of at least one coronary artery on CAG), and dyslipidemia]. White blood cell counts (WBCs) and blood chemistry tests [determination of the platelet, hemoglobin, uric acid, glucose, alanine transaminase (ALT), and creatinine levels] were performed at admission using routine laboratory methods for patients in both groups. For patients with sepsis, the C-reactive protein (CRP), procalcitonin, lactate, and albumin levels were also determined at the time of ICU admission. Information on infection sites was obtained from the descriptions of diagnoses in these patients’ medical records. Septic shock was defined as hypotension requiring vasopressor use to maintain a mean arterial pressure ≥ 65 mmHg and with a serum lactate level > 18 mg/dL [2]. Disease severity at the time of ICU admission was determined using the SOFA and Acute Physiology and Chronic Health Evaluation II (APACHE II) scores. This study was approved by the Research Ethics Committee of Taipei Veterans General Hospital (no. 2018-02-009AC) and conducted according to the principles of the Declaration of Helsinki. All participants provided written informed consent. All relevant data within this study were provided in the Supporting Information files (**S1 Dataset**).

### Determination of lipid profiles

Blood samples were collected from the participants within 24 hours of admission and stored at –80°C until analysis. Lipid profiles, including the total cholesterol, HDL-C, and triglyceride levels, were determined using routine laboratory methods. The Friedewald formula was applied to estimate the low-density lipoprotein cholesterol (LDL-C) level. The serum amyloid A (SAA) protein level was measured using the commercial Human SAA1 DuoSet ELISA Kit DY3019-05 (R&D Systems, Inc., Minneapolis, MN, USA).

### Determination of anti-inflammatory capacity in apoB-depleted plasma

The anti-inflammatory capacity of apoB-depleted plasma in each sample was determined using an *in-vitro* cell system following an established procedure [20]. For apoB-depleted plasma preparation, we mixed each sample with 36% polyethylene glycol 6000 (Merck, Darmstadt, Germany) in HEPES buffer (pH 8.0; Merck) at a 2:1 ratio and kept the mixture on ice for 30 minutes. The mixture was then centrifuged at 2200 × *g* for 30 minutes, and the supernatant (apoB-depleted plasma] was used directly for the determination of its anti-inflammatory capacity.

Human umbilical-vein endothelial cells (HUVECs; ATCC, Manassas, VA, USA) were cultured in medium with 10% fetal bovine serum (FBS). After reaching 70% confluency, the cells were incubated with FBS-free medium for 2 hours; 3% apo-B–depleted plasma or phosphate-buffered saline (control) was then added. After 30 minutes of incubation, the HUVECs were treated with 10 ng/mL TNF-α (R&D Systems) and incubated for 7 hours to stimulate VCAM-1 mRNA expression. Subsequently, RNA isolation and cDNA synthesis were performed using NucleoZol (MACHEREY-NAGEL GmbH & Co. KG, Düren, Germany) and PrimeScript RT reagent (Perfect Real Time, RR037A; Takara Bio Inc., Shiga, Japan) kits, respectively, following the manufacturers’ instructions. VCAM-1 mRNA expression was determined by quantitative real-time polymerase chain reaction (StepOnePlus; Thermo Fisher scientific, Waltham, MA, USA) using cyclophilin A as the housekeeping gene and the following primers: VCAM-1 forward, 5’-GGGAAGATGGTCGTATCCTT-3’; VCAM-1 reverse, 5’-TCTGGGGTGGTCTCGATTTTA-3’; cyclophilin A forward, 5’-TGCTGGACCCAACACAAATGGT-3’; and cyclophilin A reverse, 5’-GCCAAACACCACATGCTTGC-3’ (Thermo Fisher, Waltham, MA, USA). The fold change in gene expression was calculated using the ΔΔCt method, normalized to the corresponding control for each 12-well plate. ApoB-depleted plasma’s anti-inflammatory capacity was measured at least in duplicate and represented by the averaged fold change for each plasma sample. Larger fold changes were taken to indicate lower anti-inflammatory capacity.

### Statistical analysis

Continuous variables are presented as medians (interquartile ranges) and categorical variables are presented as numbers (percentages). The Mann–Whitney *U* test or Fisher’s exact test was used to detect between-group differences in the study variables. Multivariable logistic regression analysis was performed to control for variable imbalance and to assess whether associations of reduced anti-inflammatory capacity of apoB-depleted plasma with sepsis and septic shock were dependent on HDL-C or inflammatory markers. A subgroup analysis was conducted to investigate anti-inflammatory capacity of apoB-depleted plasma in the septic group stratified by disease severity (septic shock, lactate concentration > 18 mg/dL, or SOFA score > 8, established prognostic indicators for sepsis with clinically significant cut-off values) [2]. The Spearman coefficient was used to assess correlations of anti-inflammatory capacity of apoB-depleted plasma and HDL-C with continuous variables in the septic population. Two-tailed *p* values ≤ 0.05 were considered significant. All statistical analyses were performed with SPSS software ver. 24.0 (IBM, Armonk, NY, USA) and graph creation were performed with GraphPad Prism 8 (GraphPad Software, San Diego, CA, USA).

## Results

### Baseline participant characteristics

Totally 80 septic patients and 50 non-septic subjects admitted for CAG were included in the cross-sectional study. The clinical and laboratory variables were described in **Table 1**. Relative to the control group, patients were older, the proportion of males was larger, and the BMI was lower in the sepsis group [Age: 67.0 (60.3–79.0) vs. 63.5 (53.8–70.0), *p* = 0.0098; male gender: 60 (75.0%) vs. 26 (52.0%), *p* = 0.0082; BMI: 22.4 (19.5–26.0) vs. 24.9 (22.9–28.0), *p* = 0.0049]. In addition, the WBC, ALT and creatinine levels were higher and the hemoglobin, platelet, total cholesterol, LDL-C, and HDL-C concentrations were lower in the sepsis group [WBC: 11.3 (6.4–16.9) vs. 6.0 (4.8–7.9), *p* < 0.0001; ALT: 29.0 (15.8–49.5) vs. 16.5 (12.0–22.0), *p* < 0.0001; creatinine: 2.0 (0.9–3.5) vs. 0.9 (0.7–1.1), *p* < 0.0001; hemoglobin: 8.7 (7.8–10.9) vs. 13.3 (12.6–14.1), *p* < 0.0001; platelet: 120.5 (71.5–227.5) vs. 206.0 (181.8–250.0), *p* < 0.0001; total cholesterol: 95.0 (70.3–118.0) vs. 151.0 (113.5–182.0), *p* < 0.0001; LDL-C: 50.3 (31.0–64.2) vs. 75.2 (64.4–96.9), *p* < 0.0001; HDL-C: 18.0 (10.2–31.8) vs. 47.6 (37.1–59.6), *p* < 0.0001; **Table 1**].

**Table 1:** Baseline characteristics among septic patients and controls.

	Control (N = 50)	Sepsis (N = 80)	*P* value
Age (years)	63.5 (53.8–70.0)	67.0 (60.3–79.0)	0.0098
Male gender (%)	26 (52.0)	60 (75.0)	0.0082
Body mass index (kg/m^2^)	24.9 (22.9–28.0)	22.4 (19.5–26.0)	0.0049
Smoking history (%)	11 (22.0)	28 (35.0)	0.17
Co-morbidities (%)			
Hypertension	30 (60.0)	41 (51.3)	0.37
Type 2 diabetic mellitus	9 (18.0)	22 (27.5)	0.29
Coronary artery disease	10 (20.0)	17 (21.3)	>0.99
Atrial fibrillation	3 (6.0)	10 (12.5)	0.37
End-stage renal disease	3 (6.0)	5 (6.3)	>0.99
Gout	2 (4.0)	9 (11.3)	0.20
Dyslipidemia	23 (46.0)	8 (10.0)	<0.0001
Laboratory data			
White blood cells (K)	6.0 (4.8–7.9)	11.3 (6.4–16.9)	<0.0001
Hemoglobin (mg/dL)	13.3 (12.6–14.1)	8.7 (7.8–10.9)	<0.0001
Platelets (K)	206.0 (181.8–250.0)	120.5 (71.5–227.5)	<0.0001
ALT (U/L)	16.5 (12.0–22.0)	29.0 (15.8–49.5)	<0.0001
Creatinine (mg/dL)	0.9 (0.7–1.1)	2.0 (0.9–3.5)	<0.0001
Uric acid (mg/dL)	5.2 (4.0–5.9)	5.3 (3.2–7.8)	0.40
Total cholesterol (mg/dL)	151.0 (113.5–182.0)	95.0 (70.3–118.0)	<0.0001
HDL-C (mg/dL)	47.6 (37.1–59.6)	18.0 (10.2–31.8)	<0.0001
LDL-C (mg/dL)	75.2 (64.4–96.9)	50.3 (31.0–64.2)	<0.0001
Triglyceride (mg/dL)	122.0 (71.8–149.3)	105 (73.5–146.3)	0.29

Continuous variables were expressed as median (interquartile range) and categorical variables were expressed as number (percentage). Mann-Whitney U test or Fisher’s exact test was used to compare the variables between two groups. ALT, alanine transaminase; HDL-C, high-density lipoprotein cholesterol; LDL-C, low-density lipoprotein cholesterol

### Association between impaired anti-inflammatory capacity of apoB-depleted plasma and the presence of sepsis

**Fig 1:**
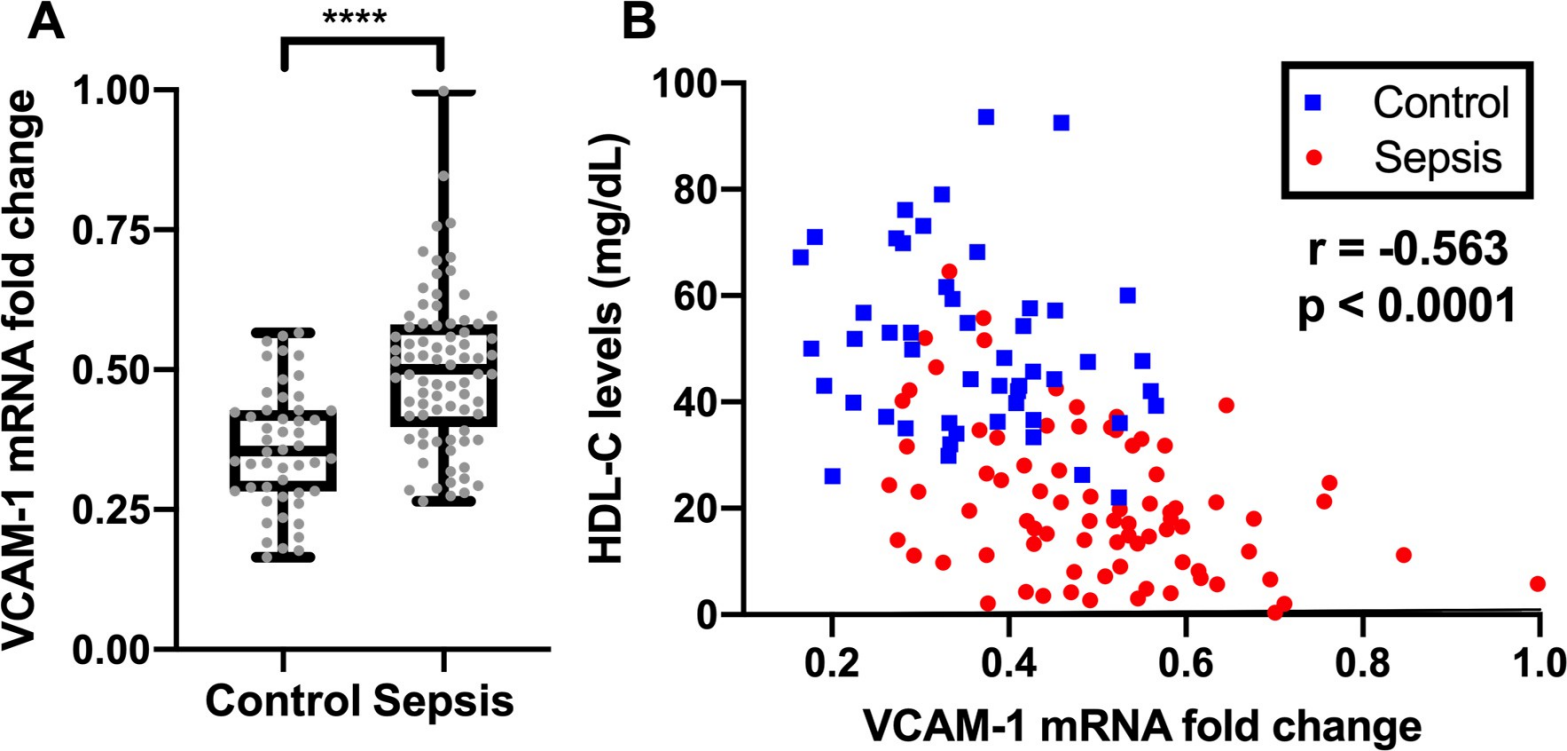
Relative expression of VCAM-1 mRNA, indicating anti-inflammatory capacity of apoB-depleted plasma. **(A)** Comparison of apoB-depleted plasma’s anti-inflammatory capacity between sepsis (n = 80) and control (n = 50). **(B)** Scatter plot showing univariate relationship between apoB-depleted plasma’s anti-inflammatory capacity and HDL-C levels in the whole population (n = 130, spearman correlation coefficient r = -0.563, *p* < 0.0001). Separating the two populations, the correlation between VCAM mRNA fold change and HDL-C is significant only in sepsis population (control: n = 50, r = -0.229, p = 0.11; sepsis: n = 80, r = -0.370; p = 0.0007).

The anti-inflammatory capacity of apoB-depleted plasma from patients with sepsis was lower than that of the control [VCAM-1 mRNA fold change: 50.1% (39.8–58.1%) vs. 35.5% (28.3–42.7%); *p* < 0.0001; **Fig 1A**]. The VCAM-1 mRNA fold change correlated well with the HDL-C level in the whole population (*r* = –0.563, *p* < 0.0001; **Fig 1B**) and the sepsis group (*r* = –0.370, *p* = 0.0007; **Fig 1B**).

ApoB-depleted plasma’s anti-inflammatory capacity was associated significantly with the presence of sepsis in a univariable logistic regression analysis {odds ratio [95% confidence interval (CI)] = 2.60 [1.82–3.93]; *p* < 0.0001}. This association remained significant with adjustment for age, sex, BMI, and dyslipidemia [odds ratio (95% CI) = 2.78 (1.78–4.36); *p* < 0.0001], but not with additional adjustment for the HDL-C level (**Table 2**, **S1 Fig**). The association between anti-inflammatory capacity and sepsis remained the same, even after excluding ten CAD subjects from control group (**S1 Table**, the left 40 control samples are with normal angiography findings, representing a relatively healthier population).

**Table 2:** Univariable and multivariable logistic regression for the association between apoB-depleted plasma’s anti-inflammatory capacity (represented by VCAM-1 mRNA fold change value) and the presence of sepsis (n = 130).

Dependent variable: sepsis	Odds ratio*	95% confidence interval	*P* value
Univariable	2.60	1.82–3.93	<0.0001
Model 1	2.88	1.88–4.41	<0.0001
Model 2	2.78	1.78–4.36	<0.0001
Model 3	1.71	0.98–2.98	0.059
Model 4	1.51	0.94–2.43	0.088

*VCAM-1 mRNA fold change values were rescaled by a factor of 10

Model 1: adjusted for age, gender, BMI

Model 2: adjusted for age, gender, BMI, Dyslipidemia

Model 3: adjusted for age, gender, BMI, Dyslipidemia, HDL-C

Model 4: adjusted for HDL-C

BMI, body mass index; HDL-C, high-density lipoprotein cholesterol

### Association between impaired apoB-depleted plasma’s anti-inflammatory capacity and the severity of sepsis

**Fig 2:**
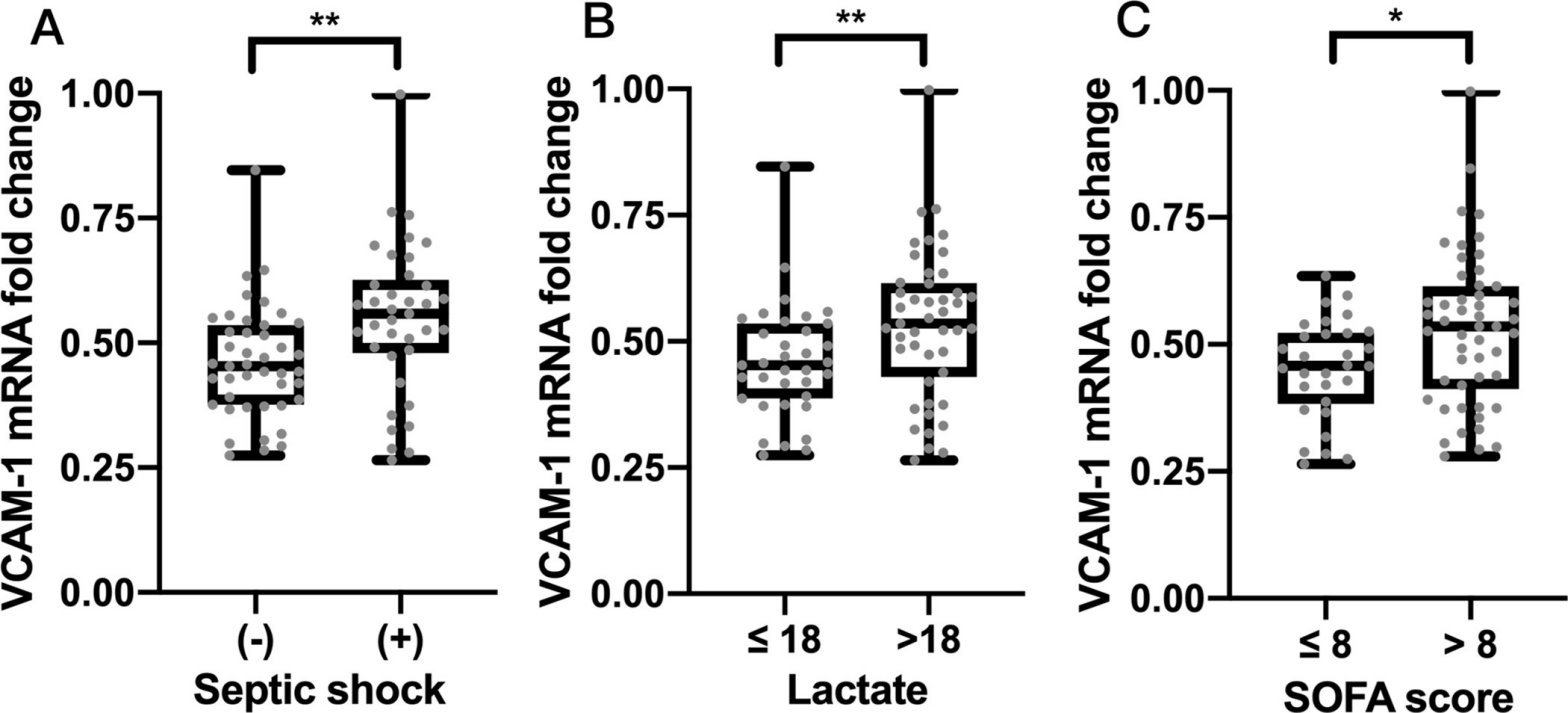
Comparison of apoB-depleted plasma’s anti-inflammatory capacity (represented as VCAM-1 mRNA fold change) between septic patients with different disease severity. **(A)** Subjects with septic shock (n = 37) *vs*. non-shock sepsis (n = 43). **(B)** Subjects with serum lactate > 18 mg/dL (n = 45) *vs*. lactate ≤ 18 mg/dL (n = 35). **(C)** Subjects with SOFA score > 8 (n = 50) *vs*. SOFA score ≤ 8 (n = 30).

Subgroup analysis was conducted to explore the association between sepsis severity and apoB-depleted plasma’s anti-inflammatory capacity. Of the patients with sepsis, 37 had septic shock and 43 did not. Their clinical characteristics and laboratory data were described in **Table 3**. Relative to patients without septic shock, those with this condition had higher SOFA and APACHE II scores and lactate and procalcitonin levels, and lower total cholesterol and HDL-C concentrations [SOFA score: 11.0 (9.0–13.0) vs. 9.0 (7.0–11.0), *p* = 0.0007; APACHE II score: 32.0 (26.5–39.5) vs. 24.0 (20.0–30.0), *p* = 0.0001; lactate level: 40.5 (25.6, 65.3) vs. 11.0 (9.1–15.4), *p* < 0.0001; procalcitonin level: 3.4 (1.1–18.9) vs. 1.0 (0.4–3.8), *p* = 0.0060; total cholesterol: 85.0 (61.5–107.0) vs. 101.0 (81.0, 128.0), *p* = 0.0056; HDL-C: 18.0 (10.2–31.8) vs. 23.5 (14.0–35.2), *p* = 0.0022; **Table 3**). ApoB-depleted plasma’s anti-inflammatory capacity (represented by the VCAM-1 mRNA fold change) was impaired more significantly in patients with greater disease severity, defined by septic shock [55.8% (48.0–62.6%) vs. 45.3% (37.6–53.5%), *p* = 0.0022; **Fig 2A**], lactate level > 18 mg/dL [53.5% (43.0–61.5%) vs. 45.3% (38.7–53.5%), *p* = 0.0076; **Fig 2B**], and SOFA score > 8 [53.5% (41.2–61.5%) vs. 45.6% (38.3%–52.3%), *p* = 0.015; **Fig 2C**]. Notably, the distributions of apoB-depleted plasma’s anti-inflammatory capacity were sparser for these three subgroups than for their counterparts with less-severe disease.

**Table 3:** Clinical and laboratory characteristics among septic subjects without and with septic shock.

	Non-shock sepsis (N = 43)	Septic shock (N = 37)	*P* value
Age (years)	69.0 (62.0–83.0)	67.5 (55.5–77.5)	0.15
Male gender (%)	33 (76.3)	27 (70.3)	0.80
Body mass index (kg/m^2^)	23.1 (19.5–26.1)	22.2 (19.7–25.9)	>0.99
Smoking history (%)	19 (44.2)	9 (24.3)	0.099
SOFA score	9.0 (7.0–11.0)	11.0 (9.0–13.0)	0.0007
APACHE II score	24.0 (20.0–30.0)	32.0 (26.5–39.5)	0.0001
Infection sites (%)			
Pneumonia	28 (65.1)	22 (59.5)	0.65
Urinary tract infection	4 (9.3)	4 (10.8)	>0.99
Blood-stream infection	3 (7.0)	3 (8.1)	>0.99
Intraabdominal infection	8 (18.6)	6 (16.2)	>0.99
Soft tissue infection	5 (11.6)	2 (5.4)	0.44
CNS infection	0 (0)	1 (2.7)	0.46
Co-morbidities (%)			
Hypertension	25 (58.1)	16 (43.2)	0.26
Type 2 diabetic mellitus	13 (30.2)	9 (24.3)	0.62
Coronary artery disease	10 (23.3)	7 (19.0)	0.76
Atrial fibrillation	5 (11.2)	5 (13.5)	>0.99
End-stage renal disease	3 (7.0)	2 (5.4)	>0.99
Gout	6 (14.0)	3 (8.1)	0.49
Dyslipidemia	6 (14.0)	2 (5.4)	0.28
Laboratory data			
White blood cells (K)	9.2 (5.5–13.5)	12.8 (6.5–19.0)	0.077
Hemoglobin (mg/dL)	9.2 (7.7–11.1)	8.5 (7.9–9.7)	0.23
Platelets (K)	140.0 (77.0–242.2)	98 (49–206)	0.16
ALT (U/L)	29.0 (15.0–39.0)	29.0 (16.0–91.0)	0.42
Creatinine (mg/dL)	1.55 (0.8–4.0)	2.5 (1.0–3.5)	0.42
Uric acid (mg/dL)	4.4 (3.0–7.0)	6.6 (4.3–9.9)	0.023
Total cholesterol (mg/dL)	101.0 (81.0, 128.0)	85.0 (61.5–107.0)	0.0056
HDL-C (mg/dL)	23.5 (14.0–35.2)	18.0 (10.2–31.8)	0.0022
LDL-C (mg/dL)	55.3 (37.3–74.1)	46.1 (23.0–63.2)	0.052
Triglyceride (mg/dL)	102.0 (76.0–141.0)	107.0 (57.0–150.0)	0.67
Albumin (mg/dL)	3.0 (2.7–3.6)	2.9 (2.5,3.2)	0.095
Lactate (mg/dL)	11.0 (9.1–15.4)	40.5 (25.6, 65.3)	<0.0001
CRP (mg/dL)	12.0 (5.9–16.7)	15.9 (7.2–24.7)	0.052
Procalcitonin (ng/dL)	1.0 (0.4–3.8)	3.4 (1.1–18.9)	0.0060
Serum amyloid A (mg/dL)	70.7 (36.1–140.7)	73.3 (8.4, 164.3)	0.25

Continuous variables were expressed as median (interquartile range) and categorical variables were expressed as number (frequency percentage). Mann-Whitney U test or Fisher’s exact test was used to compare the variables between two groups. CNS, central nervous system; ALT, alanine transaminase; HDL-C, high-density lipoprotein cholesterol; LDL-C, low-density lipoprotein cholesterol; CRP, c-reactive protein

The association between the impairment of apoB-depleted plasma’s anti-inflammatory capacity and the presence of septic shock was significant in multivariable logistic analyses adjusted for the WBC [odds ratio (95% CI) = 1.54 (1.03–2.29), *p* = 0.035], CRP level [odds ratio (95% CI) = 1.53 (1.04–2.26), *p* = 0.030], procalcitonin level [odds ratio (95% CI) = 1.68 (1.12–2.51), *p* = 0.012], and SAA level [odds ratio (95% CI) = 1.68 (1.15–2.46), *p* = 0.0078], but not in an analysis adjusted for the HDL-C level [odds ratio (95% CI) = 1.43 (0.95–2.14), *p* = 0.087; **Table 4**, **S2 Fig**]. These results suggest that the impairment of apoB-depleted plasma’s anti-inflammatory capacity during sepsis was dependent on the HDL-C concentration.

**Table 4:** Univariable and multivariable logistic regression for the association between apoB-depleted plasma’s anti-inflammatory capacity and the presence of septic shock (n = 80).

Dependent variable: septic shock	Odds ratio*	95% confidence interval	*P* value
Univariable	1.68	1.15–2.47	0.0078
Model 1	1.68	1.15–2.46	0.0078
Model 2	1.68	1.12–2.51	0.012
Model 3	1.53	1.04–2.26	0.030
Model 4	1.54	1.03–2.29	0.035
Model 5	1.43	0.95–2.14	0.087

*VCAM-1 mRNA fold change values were rescaled by a factor of 10

Model 1: adjusted for serum amyloid A

Model 2: adjusted for procalcitonin

Model 3: adjusted for C-reactive protein

Model 4: adjusted for white blood cell

Model 5: adjusted for HDL-C

HDL-C, high-density lipoprotein cholesterol

### Correlations of apoB-depleted plasma’s anti-inflammatory capacity and HDL-C concentration with clinical variables

In septic population, the correlations between VCAM-1 mRNA fold change and other continuous variables were presented in **Table 5**. Consistent with the result obtained for the whole population, the HDL-C concentration correlated positively with the VCAM-1 mRNA fold change in the sepsis group (*r* = 0.370, *p* = 0.0007). This fold change also correlated moderately with the CRP level (*r* = 0.441, *p* < 0.0001) and WBC (*r* = 0.353, *p* = 0.0013), and with the SOFA score (*r* = 0.231, *p* = 0.039), lactate level (*r* = 0.297, *p* = 0.0074), and procalcitonin level (*r* = 0.258, *p* = 0.025; **Table 5**). The HDL-C concentration correlated with disease severity indicators (SOFA score: *r* = –0.435, *p* < 0.0001; lactate level: *r* = –0.378, *p* = 0.0005; APACHE II score: *r* = –0.243, *p* = 0.030) and inflammatory markers (procalcitonin level: *r* = 0.444, *p* < 0.0001; CRP level: *r* = –0.431, *p* < 0.0001). Neither the HDL-C concentration nor VCAM-1 mRNA fold change correlated significantly with the SAA level.

**Table 5:** Correlations of clinical variables with apoB-depleted plasma’s anti-inflammatory capacity (represented as VCAM-1 mRNA fold change value) or HDL-C among septic patients (n = 80).

	With VCAM-1 mRNA FC value With HDL-C			
	Coefficient (r)	*P*	Coefficient (r)	*P*
Age	0.070	0.54	-0.147	0.20
Body mass index	0.140	0.22	-0.168	0.14
APACHE II score	0.137	0.23	-0.243	0.030
SOFA score	0.231	0.039	-0.435	<0.0001
Lactate	0.297	0.0074	-0.378	0.0005
Total cholesterol	-0.341	0.0019	0.571	<0.0001
HDL-C	-0.370	0.0007	-	-
LDL-C	-0.308	0.0055	0.454	<0.0001
Triglyceride	0.134	0.24	-0.504	<0.0001
Procalcitonin	0.258	0.025	-0.444	<0.0001
C-reactive protein	0.441	<0.0001	-0.438	<0.0001
Serum amyloid A	0.052	0.65	-0.136	0.23
White blood cells	0.353	0.0013	-0.149	0.19
Hemoglobin	-0.137	0.22	0.060	0.60
Platelets	0.004	0.97	0.236	0.035
Creatinine	-0.027	0.81	-0.077	0.50
Alanine transaminase	-0.079	0.49	-0.078	0.49
Albumin	-0.279	0.012	0.321	0.0037
Uric acid	-0.051	0.66	0.003	0.98

FC, fold change; APACHE II, acute physiology and chronic health evaluation II; SOFA, sequential organ failure assessment; LDL-C, low-density lipoprotein cholesterol; HDL-C, high-density lipoprotein cholesterol

## Discussion

In this cross-sectional study, we demonstrated that the anti-inflammatory capacity of apoB-depleted plasma was significantly reduced in patients with sepsis, and that this reduction was more pronounced in patients with than in those without septic shock and depended on the HDL-C concentration. Moreover, the anti-inflammatory capacity of apoB-depleted plasma correlated with the SOFA score, lactate level, and inflammatory markers in the septic population. These findings improve our understanding of the role of HDL in sepsis. HDL and its anti-inflammatory capacity could be novel prognostic markers, or even treatment targets, for sepsis.

### Sepsis-associated impairment of HDL’s anti-inflammatory capacity

The term “anti-inflammatory capacity” refers to HDL’s ability to inhibit TNF-α–induced VCAM-1 expression in endothelial cells. Several diseases featuring chronic or acute inflammation, including acute myocardial infarction (AMI), T2DM, AF and nonalcoholic fatty live disease, have been reported to associate with the impairment of HDL’s anti-inflammatory capacity [19–22]. This impairment causes the loss of HDL’s vasoprotective effect and contributes to endothelial dysfunction, facilitating systemic inflammation and tissue impairment in sepsis [8, 17, 23]. A previous study revealed an overall property transition of HDL isolated from patients with septic acute respiratory distress syndrome [24]; the present work had a narrower measurement target, enabling focused investigation of the clinical relevance of HDL’s functionality.

In the present study, the association between impaired apoB-depleted plasma’s anti-inflammatory capacity and the presence of sepsis depended on the HDL-C level. In addition, the anti-inflammatory capacity correlated moderately with the HDL-C level in the whole population and sepsis group, but not the control group. These observations are important given emerging evidence, such as that from the REVEAL clinical trial [25], suggesting that the HDL-C level is not necessarily indicative of the functional capacity of HDL particles [15, 25–28]. The revealed dependency may explain the prognostic role of the HDL-C index in sepsis [10] and indirectly supports the rationale for the targeting of the HDL-C level in the treatment of sepsis [12]. A CETP inhibitor that prevented HDL from exchanging its cholesterol component for triglycerides carried by non-HDL lipoprotein was found to improve the survival of septic mice [12]. The decreased CETP activity observed in sepsis is regarded as a compensatory mechanism preventing systemic endotoxemia [14, 29]. A CETP inhibitor was hypothesized to attenuate HDL-C loss and help to preserve functionality of HDL particles during sepsis. Although this agent was not found to contribute significantly to the reduction of the incidence of cardiovascular disease by elevated HDL-C level in recent trials [25], whether a CETP inhibitor can improve the prognosis of sepsis in humans is worth investigating, as the decline of the HDL-C level is more pathologically striking and appears to be combined with decreased functional metrics in sepsis [23, 30, 31].

### Association of the impairment of HDL anti-inflammatory capacity with sepsis severity

We found that apoB-depleted plasma’s anti-inflammatory capacity was impaired more strongly in patients with more severe disease (defined as septic shock, SOFA score > 8, and lactate level > 18 mg/dL) [2]. Consistently, the association of the anti-inflammatory capacity with septic shock depended on the HDL-C level. Although the temporality of the association between the impairment of anti-inflammatory capacity and greater disease severity could not be examined in this cross-sectional study, our findings raise some intriguing points worth discussing. First, the association of the SOFA score (which reflects organ dysfunction) [32], but not the APACHE II score, with apoB-depleted plasma’s anti-inflammatory capacity seems to be reasonable because the regulation of endothelial VCAM-1 expression plays an important role in the avoidance of shock and tissue damage during sepsis [3, 17]. Second, the distribution of apoB-depleted plasma’s anti-inflammatory capacity was sparser (reflecting more intragroup variation) for patients with more severe disease (i.e. septic shock, SOFA > 8, lactate > 18mg/dL). The heterogeneity nature of sepsis was amplified in subgroups with higher severity. In an RNA sequencing–based classification [33], most patients with sepsis and poor prognoses were classified as having strong inflammation or neutrophilic suppression endotypes, which have very different genetic profiles. The dysregulation of the host response varies bipolarly among septic subjects and may influence HDL’s composition and functions in different ways [3, 33, 34]. More cautious, subtype-based, methods of sepsis classification should be applied in future work.

### HDL’s anti-inflammatory capacity as a potential biomarker in sepsis

HDL dysfunctionality is known to result from compositional remodeling related to inflammation [35, 36]. Proteome alterations of HDL during sepsis have been described [37, 38]. In our study, the CRP level and WBC, generic inflammatory markers, correlated inversely with apoB-depleted plasma’s anti-inflammatory capacity in patients with sepsis, consistent with the concept of inflammation-induced HDL dysfunction. However, no such correlation was observed for the plasma level of SAA, regarded as the culprit for apolipoprotein-AI replacement in HDL particles [39, 40]. The plasma SAA level in septic subjects may be too high to have distinguishable effects on HDL’s anti-inflammatory capacity. As we did not examine the composition of HDL, we could not determine whether the plasma SAA level was proportionate to the accumulation of SAA in HDL species. Additionally, a recent study found no association between SAA and 28-day mortality in a sepsis population, while other markers such as activity of HDL-associated paraoxonase-1 and lecithin-cholesterol acyltransferase activity displayed promising prognostic potentials [41, 42]. Seemingly, although SAA becomes predominant in the proteome of acute phase HDL, it is not necessarily the key determinant of HDL functionality.

As apoB-depleted plasma’s anti-inflammatory capacity has been shown to have added value relative to the HDL-C level for the prediction of incident cardiovascular events in general and post-AMI populations [15, 43], we explored this metrics’ potential as a sepsis biomarker by evaluating its correlations with clinical variables. It did not correlate more strongly than the HDL-C level, an existing prognostic factor for sepsis [10], with the SOFA or APACHE II scores or laboratory parameters except the WBC, indicating that this functional index may have no added value. From the prospective of pathogenesis, the decline in the circulatory HDL-C level is related to endotoxin clearance, which is probably a more critical core event in sepsis [11, 12, 44, 45]. However, an examination of the usefulness of the HDL inflammatory index (i.e., HDL’s ability to protect LDL from oxidization) as a sepsis biomarker showed that a dynamic change in HDL functionality is more useful in the evaluation of prognosis because it reflects the recovery or deterioration of patients’ physiological status [46]. As we performed only static measurement at a single timepoint in this study, further investigation is needed to determine whether HDL’s anti-inflammatory capacity is more informative than the HDL-C level about the prognosis of sepsis.

### Limitations

This study has several limitations. First, it was conducted with a small sample size at a single center. Given the heterogeneity of lipid profiles among people of different ethnicities [47, 48], our findings may not be applicable to other populations. Second, demographic differences existed between the patients with sepsis and those admitted for CAG. Although we adjusted for these variables in the multivariable logistic regression analysis, residual confounding may have existed. Third, due to the cross-sectional study design, we could not infer causal relationships. In addition, the prognostic value of HDL’s anti-inflammatory capacity was not explored completely, as sepsis outcomes and prospective parameters were not examined. Fourth, as HDL is versatile and has pleiotropic effects on endothelial cells, macrophages, smooth muscle cells, etc., there are many other assays of HDL anti-inflammatory functions [16]. Our work examined only one aspect of HDL anti-inflammatory functions and was thus limited to the assay we have done. Finally, it should be noted that we use apoB-depleted plasma, which does not equal purely isolated HDL, to measure HDL anti-inflammatory capacity. In contrast to ultracentrifugation, the method that we used for HDL preparation preserves not only HDL particles but also few other unbounded proteins in plasma [49]. Although, according to previous study, 83% of the biological effect of apoB–depleted plasma is attributed to HDL particles [15], this is not necessarily the case in acute phase samples. The cost-effectiveness of apo-B depletion allows for a relatively larger sample size in researching on HDL functionality. Due to the concern that the measurements were biased by the high amount of inflammatory cytokines presented in septic samples [50], we had checked interleukin-6 (IL-6) levels in 12 selected samples by ELISA kits (R&D Systems, Inc., Minneapolis, MN, USA). The highest IL-6 level among those samples (75 pg/mL in cell cultures) was a lot lower than 10 ng/mL TNF-α added following our experimental protocol (**S2 Table**). We found no correlation trend (spearman’s r = 0.021) between VCAM-1 mRNA fold change value and IL-6 level in these samples (**S2 Table**). Further studies are needed to compare the anti-inflammatory capacity of purely isolated HDL and apoB-depleted plasma in sepsis.

## Conclusion

This study showed that apoB-depleted plasma’s anti-inflammatory capacity is significantly reduced in patients with sepsis, and that this association depends on the HDL-C concentration. In patients with sepsis, apoB-depleted plasma’s anti-inflammatory capacity correlates with disease severity and various inflammatory markers, such as the CRP level and WBC. These findings link HDL functionality to clinical variables, provide an explanation for the prognostic role of the HDL-C level in sepsis, and indirectly support the rationale for HDL-C targeting as a sepsis treatment.

## Supporting information

S1 Fig Conversion of Table 2 into figure type. Univariable and multivariable logistic regression for the association between apoB-depleted plasma’s anti-inflammatory capacity (represented by VCAM-1 mRNA fold change value) and the presence of sepsis (n = 130). *VCAM-1 mRNA fold change values were rescaled by a factor of 10. Model 1: adjusted for age, gender, BMI. Model 2: adjusted for age, gender, BMI, Dyslipidemia. Model 3: adjusted for age, gender, BMI, Dyslipidemia, HDL-C. Model 4: adjusted for HDL-C.
(TIF)

S2 Fig Conversion of Table 4 into figure type. Univariable and multivariable logistic regression for the association between apoB-depleted plasma’s anti-inflammatory capacity and the presence of septic shock (n = 80). *VCAM-1 mRNA fold change values were rescaled by a factor of 10.
(TIF)

S1 Dataset All relevant data are within the paper and its supporting files. (XLSX)

S1 Table Re-analysis excluding the 10 samples diagnosed with CAD in control population (n = 120). (DOCX)

S2 Table Interleukin-6 levels in selected 12 plasma sample before and after apo-B depletion. (DOCX)

## List of abbreviations

HDL: high-density lipoprotein
HDL-C: high-density lipoprotein cholesterol
VCAM-1: vascular cell adhesion molecule-1
BMI: body mass index
SOFA: sequential organ failure assessment
CETP: cholesteryl ester transfer protein
TNF-α: tumor necrosis factor-α
APACHE II: acute physiology and chronic health evaluation II
ICU: intensive care unit
CAG: coronary angiography
T2DM: type 2 diabetes mellitus
AF: atrial fibrillation
WBC: white blood cell
CRP: c-reactive protein
ALT: alanine transaminase
LDL-C: low-density lipoprotein cholesterol
SAA: serum amyloid A
HUVEC: human umbilical-vein endothelial cells
FBS: fetal bovine serum
apoB-depleted: apolipoprotein-B depleted
CI: confidence interval
AMI: acute myocardial infarction
