# Supplementary figures and images for: The high-density lipoprotein cholesterol (HDL-C)-concentration-dependent association between anti-inflammatory capacity and sepsis: A single-center cross-sectional study
Source: PLoS One. 2024 Apr 11;19(4):e0296863. doi: 10.1371/journal.pone.0296863 (PMC11008828; doi:10.1371/journal.pone.0296863)

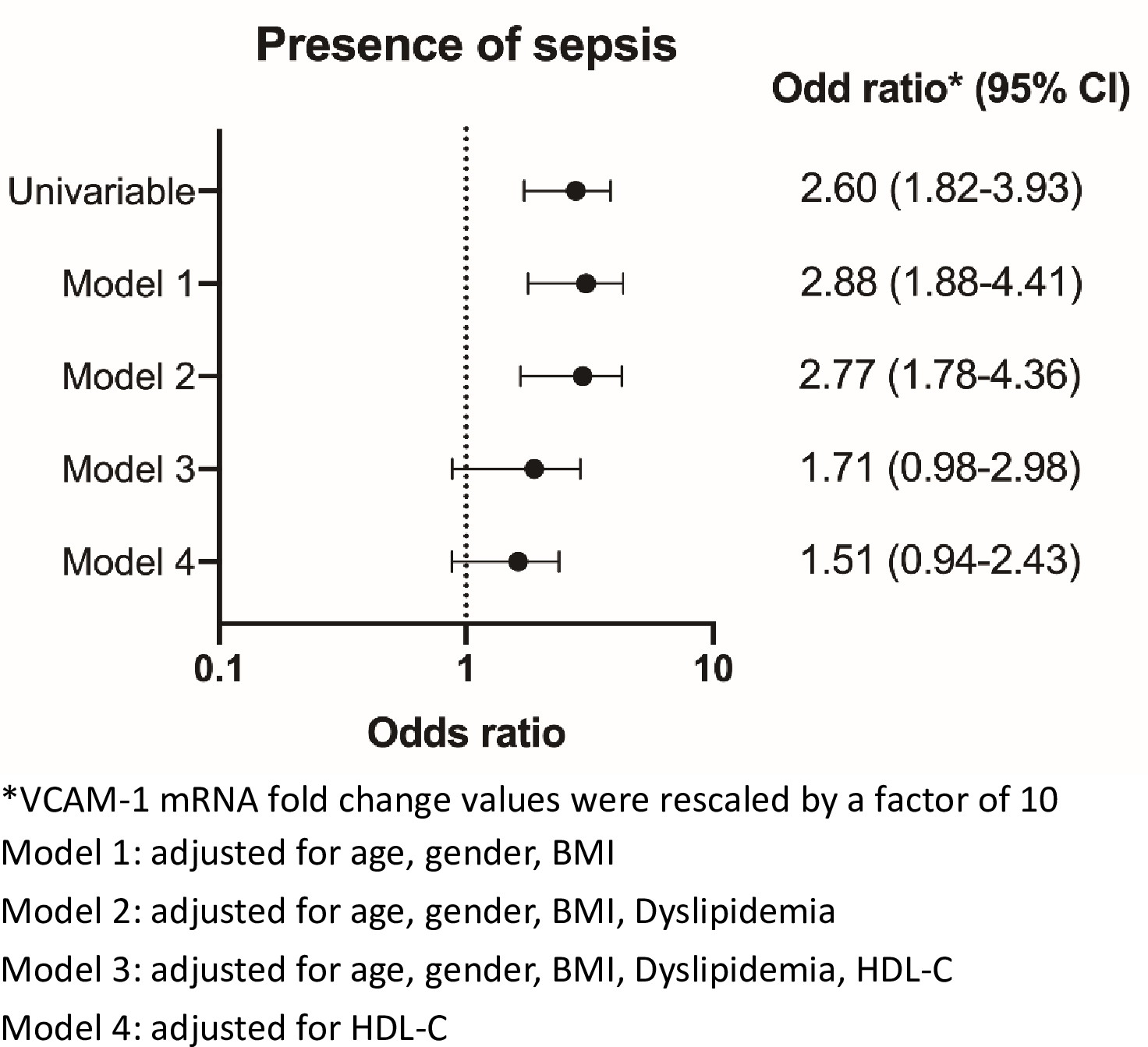

Supplement: S1 Fig — Univariable and multivariable logistic regression for the association between apoB-depleted plasma’s anti-inflammatory capacity (represented by VCAM-1 mRNA fold change value) and the presence of sepsis (n = 130). *VCAM-1 mRNA fold change values were rescaled by a factor of 10. Model 1: adjusted for age, gender, BMI. Model 2: adjusted for age, gender, BMI, Dyslipidemia. Model 3: adjusted for age, gender, BMI, Dyslipidemia, HDL-C. Model 4: adjusted for HDL-C. (TIF) [file pone.0296863.s001.tif]

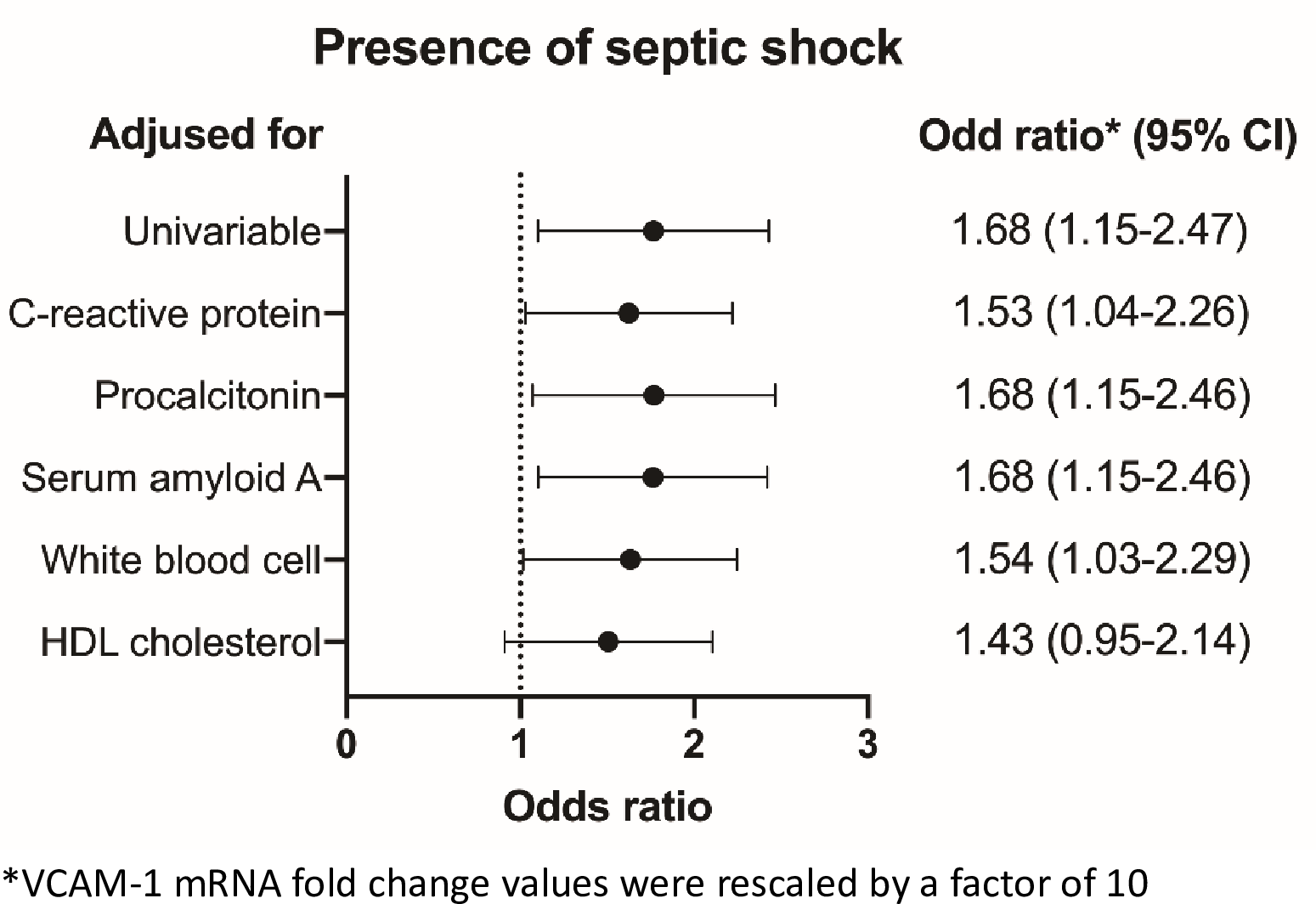

Supplement: S2 Fig — Univariable and multivariable logistic regression for the association between apoB-depleted plasma’s anti-inflammatory capacity and the presence of septic shock (n = 80). *VCAM-1 mRNA fold change values were rescaled by a factor of 10. (TIF) [file pone.0296863.s002.tif]
