# Supplementary material for: The high-density lipoprotein cholesterol (HDL-C)-concentration-dependent association between anti-inflammatory capacity and sepsis: A single-center cross-sectional study
Source: PLoS One. 2024 Apr 11;19(4):e0296863. doi: 10.1371/journal.pone.0296863 (PMC11008828; doi:10.1371/journal.pone.0296863)
Supplement: S1 Table — (DOCX) [file pone.0296863.s004.docx]

**S1 Table.** **Univariable and multivariable logistic regression for the association between apoB-depleted plasma’s anti-inflammatory capacity (represented by VCAM-1 mRNA fold change value) and the presence of sepsis (n=120).**

| **Dependent variable: sepsis** | **Odds ratio*** | **95% confidence interval** | ***P* value** |
| --- | --- | --- | --- |
| Univariable | 2.39 | 1.61-3.57 | <0.0001 |
| Model 1 | 2.37 | 1.53-3.69 | <0.0001 |
| Model 2 | 2.59 | 1.65-4.05 | <0.0001 |
| Model 3 | 1.25 | 0.68-2.31 | 0.437 |
| Model 4 | 1.16 | 0.68-1.98 | 0.595 |

* VCAM-1 mRNA fold change values were rescaled by a factor of 10

Model 1: adjusted for age, gender, BMI

Model 2: adjusted for age, gender, BMI, Dyslipidemia

Model 3: adjusted for age, gender, BMI, Dyslipidemia, HDL-C

Model 4: adjusted for HDL-C

BMI, body mass index; HDL-C, high-density lipoprotein cholesterol
