# Supplementary material for: The high-density lipoprotein cholesterol (HDL-C)-concentration-dependent association between anti-inflammatory capacity and sepsis: A single-center cross-sectional study
Source: PLoS One. 2024 Apr 11;19(4):e0296863. doi: 10.1371/journal.pone.0296863 (PMC11008828; doi:10.1371/journal.pone.0296863)
Supplement: S2 Table — (DOCX) [file pone.0296863.s005.docx]

**S2 Table.** **Interleukin-6 levels in selected 12 samples.**

| **Sample**  **NO.** | **IL-6 level in plasma sample**  **(pg/mL)** | **IL-6 level in apoB-depleted plasma (pg/mL)** | **HDL-C level**  **(mg/dL)** | **VCAM-1 mRNA fold change** |
| --- | --- | --- | --- | --- |
| H1 | Out of range | 43.9 | 39.3 | 0.566 |
| H41 | Out of range | 18.5 | 35.0 | 0.283 |
| H42 | Out of range | 37.5 | 36.0 | 0.333 |
| H43 | Out of range | 29.0 | 53.0 | 0.289 |
| S71 | 173.8 | 162.0 | 24.4 | 0.265 |
| S72 | 2398.0 | 2278.0 | 40.2 | 0.280 |
| S75 | 64.8 | 96.2 | 17.6 | 0.420 |
| S76 | 88.2 | 97.9 | 24.8 | 0.762 |
| S77 | 19.1 | 44.8 | 2.0 | 0.711 |
| S78 | 61.3 | 76.0 | 8.2 | 0.614 |
| S79 | 2091.0 | 1937.4 | 14.7 | 0.558 |
| S80 | 2538.7 | 2536.9 | 17.1 | 0.535 |
